# Explaining individual differences in infant visual sensory seeking

**DOI:** 10.1111/infa.12356

**Published:** 2020-08-03

**Authors:** Elena Serena Piccardi, Mark H. Johnson, Teodora Gliga

**Affiliations:** ^1^ Centre for Brain and Cognitive Development Birkbeck University of London London UK; ^2^ Department of Psychology Cambridge University Cambridge UK; ^3^ Department of Psychology University of East Anglia Norwich UK

## Abstract

Individual differences in infants’ engagement with their environment manifest early in development and are noticed by parents. Three views have been advanced to explain differences in seeking novel stimulation. The *optimal stimulation hypothesis* suggests that individuals seek further stimulation when they are under‐responsive to current sensory input. The *processing speed hypothesis* proposes that those capable of processing information faster are driven to seek stimulation more frequently. The *information prioritization hypothesis* suggests the differences in stimulation seeking index variation in the prioritization of incoming relative to ongoing information processing. Ten‐month‐old infants saw 10 repetitions of a video clip and changes in frontal theta oscillatory amplitude were measured as an index of information processing speed. Stimulus‐locked P1 peak amplitude in response to checkerboards briefly overlaid on the video at random points during its presentation indexed processing of incoming stimulation. Parental report of higher visual seeking did not relate to reduced P1 peak amplitude or to a stronger decrease in frontal theta amplitude with repetition, thus not supporting either the *optimal stimulation* or the *processing speed hypotheses*. Higher visual seeking occurred in those infants whose P1 peak amplitude was greater than expected based on their theta amplitude. These findings indicate that visual sensory seeking in infancy is explained by a bias toward novel stimulation, thus supporting the *information prioritization* hypothesis.

**Research highlights**

- This study combines parent‐reported and experimental measures to investigate the nature of individual differences in infant sensory seeking.
- Theta oscillatory amplitude, indexing learning progress, and VEPs to sudden‐onset checkerboards, indexing responsiveness to sensory input, are measured to test three hypotheses.
- Higher parent‐reported visual seeking associates with a stronger bias to prioritize incoming over ongoing information processing, but not with decreased responsiveness to sensory input or faster learning.
- We provide an objective marker of individual differences in visual sensory seeking in infancy.

## 1 INTRODUCTION

Infants’ sensory environment is complex and cluttered, containing many competing inputs to which attention can be allocated. The ability to deploy attention to relevant stimuli is one of the first coordinated active exploration abilities to emerge in development (Amso & Scerif, 2015) and is a gateway for learning and memory (Posner, 2011). Even before they can ask questions, infants manifest differences in how actively they engage with their environment. Observational studies, in which infants’ exploration of their environment is recorded, describe variation in how many of the objects in their proximity or how many different aspects of a complex object infants engage with (Bornstein, Hahn, & Suwalsky, 2013; Muentener, Herrig & Schulz, 2018). Similarly, differences manifest in infants’ propensity to approach novel stimuli (Lakatos et al., 2003). Studies using parent‐reported questionnaires, such as the Infant‐Toddler Sensory Profile (ITSP) (Dunn, 2002), capture differences in the extent to which infants are driven toward novel stimulation, for example by asking how much the child enjoys looking at shiny or moving objects or at fast‐paced TV shows.

Different theoretical proposals have been put forward to explain individual differences in seeking novel stimulation. According to one theoretical view, individuals’ active engagement with their environment strives to achieve an optimal level of stimulation (Zentall & Zentall, 1983). For example, it was suggested that decreased seeking of stimulation develops as a strategy to protect an organism that is either exposed to intense stimulation or that responds too strongly to sensory input. This proposal draws heavily on studies of sensory processing in atypical populations. Sensory atypicalities, manifested as increased or decreased sensitivity or as atypical seeking of sensory stimulation, are reported in Autism Spectrum Disorders (ASD) (Ben‐Sasson et al., 2009; Damiano‐Goodwin et al., 2018; Mulligan & White, 2012), Attention Deficit‐Hyperactivity Disorder (ADHD) (Bijlenga, Tjon‐Ka‐Jie, Schuijers, & Kooij, 2017; Dunn & Bennett, 2002; Ghanizadeh, 2011; Yochman, Parush, & Ornoy, 2004), pre‐term birth (Beranova et al., 2017; Bröring et al., 2018), and Fragile X syndrome (Baranek et al., 2002; Rogers, Hepburn, & Wehner, 2003). For example, during early childhood, ASD has often been associated with increased behavioral (Baranek, Boyd, Poe, David, & Watson, 2007; Baranek, Foster, & Berkson, 1997) and neural response to sensory input (Kolesnik et al., 2019; Miyazaki et al., 2007), and decreased seeking of sensory stimulation (Beranova et al., 2017; Mulligan & White, 2012; Ben‐Sasson et al., 2009; Tomchek & Dunn, 2007; but see Damiano‐Goodwin et al., 2018). Conversely, during late childhood and adulthood, ASD has been linked to both increased and decreased behavioral (Ausderau et al., 2014; Rogers & Ozonoff, 2005) and neural response to sensory input (Cascio, Gu, Schauder, Key, & Yoder, 2015; Marco, Hinkley, Hill, & Nagarajan, 2011), and elevated seeking of restricted, repetitive, and often self‐produced sensory stimulation (Ben‐Sasson et al., 2009; Lane, Young, Baker, & Angley, 2010; Liss, Saulnier, Fein, & Kinsbourne, 2006; Simpson, Adams, Alston‐Knox, Heussler, & Keen, 2019; Tomchek, Little, Myers, & Dunn, 2018).

Increased or decreased sensitivity and atypical seeking of sensory stimulation have mostly been investigated separately in individuals with atypical development, but Donkers et al. (2015) reported that smaller amplitude of evoked potentials to auditory input associated with increased sensory seeking in 4–12 years old children with ASD—a result aligning to the optimal stimulation hypothesis. No studies have yet assessed this theoretical claim in typically developing infants.

Others have proposed that individual differences in seeking stimulation may reflect differences in information processing abilities. Models of attention concur in suggesting that information is foraged for in a similar way as other resources (e.g., food), where a current source of information is sampled (exploited) until the effort needed to extract additional information outweighs the effort needed to seek information (explore) elsewhere, at which point a shift in the direction of attention occurs (Calhoun & Hayden, 2015; Hills, Todd, Lazer, Redish, & Couzin, 2015). Thus, it follows that the faster individuals process information, the more different sources of information they may be able to seek, and process. From a developmental perspective, information processing speed has been proposed as a factor underlying cognitive continuity from infancy to childhood (Colombo, 1993). Indeed, early observational measures of object exploration (e.g., the number of objects infants touched and the duration of object manipulation), which can be conceived as an index of seeking perceptual novelty, associate with childhood measures of IQ (Banerjee & Tamis‐LeMonda, 2007; Bornstein et al., 2013). Despite this evidence, it remains a question for debate whether cognitive ability drives the seeking of novel sensory input (Powell & Nettleback, 2014; Von Stumm, Hell, & Chamorro‐Premuzic, 2011).

Finally, a third theoretical proposal suggests that, rather than reflecting differences in information processing, differences in seeking novel stimulation are a marker of individual variation in the prioritization of incoming relative to ongoing information processing (Desimone & Duncan, 1995). While a shift between exploitation and exploration is expected as a current source of information is depleted (i.e., the information is learned) (Cohen, McClure, & Yu, 2007), exactly how much learning is considered sufficient to disengage with a current stimulus, when the opportunity to engage with novel stimulation appears, is subject to individual variation. Infants’ approach of novel objects is under the influence of dopamine receptor polymorphisms (Lakatos et al., 2003), suggesting that prioritization of novel stimulation may be done by assigning it a reward value (Snyder, Blank, & Marsolek, 2008).

In development, habituation studies have been used to capture attention shifting from familiar (ongoing) to unfamiliar (incoming) information. In a classical habituation design, infants are presented with repeated stimulation, such as a repeated image, either on its own, or paired with a stimulus that changes from trial to trial (see Colombo & Mitchell, 2009 for a review). A pattern of sustained, followed by decreasing, look durations to a central stimulus is believed to reflect initial encoding of stimulus properties and subsequent depletion of information, once encoded (Hunter & Ames, 1988). When familiar and unfamiliar stimuli are presented side by side, an initial preference for the repeated but incompletely encoded stimulus is followed by a shift of looking to the changing stimulus (Fantz, 1964; Roder, Bushnell, & Sasseville, 2000; Rose & Feldman, 1987). However, individual variation in how fast infants’ looking shifts away from the repeated stimulus was either interpreted to index processing speed (e.g., Colombo, Mitchell, Coldren, & Freeseman, 1991) or differences in seeking perceptual novelty (e.g., Gottlieb, Oudeyer, Lopes, & Baranes, 2013), for the reason that looking behavior is dependent on both these factors, that is, on how fast a stimulus is processed and on the relative value given to the information remaining to be learned versus novel information. To tease apart the two processes and investigate the mechanisms underlying individual differences in seeking novel stimulation, we need to use separate indices of learning progress and stimulus selection. We develop such measures in the current study.

Modulations of the frontal EEG theta oscillations have been shown to index information encoding in both adults (Klimesch, 1999) and infants (Begus, Southgate, & Gliga, 2015, 2016; Orekhova, Stroganova, Posikera, & Elam, 2006). For example, oscillations in the frontal theta band during object manipulation predicted infants’ subsequent object memory (Begus, Southgate, & Gliga, 2015). Sustained frontal theta power was linked to the initial phase of learning and declined, as adult participants improved performance (Clarke, Roberts, & Ranganath, 2018). In the current study, we measured theta oscillatory amplitude as an index of information processing progress, in a design which involved presenting a video stimulus repeatedly. We predict that, as infants progress through the repetitions of the video clip, a pattern of sustained followed by decrease in frontal theta oscillatory amplitude will manifest. This non‐linear modulation would reflect the progressive encoding and depletion of information (Nordt, Hoehl, & Weigelt, 2016). In addition to measuring video‐locked theta amplitude, we measured visual‐evoked potentials (VEPs), in response to briefly presented checkerboard stimuli, randomly interrupting the movie, as an index of processing incoming stimulation. We predict that we will observe a reverse profile of modulation of the early sensory component P1 (primarily capturing feedforward visual processing; Luck, 2014), which will inversely relate to theta oscillatory amplitude. This design resembles the “interrupted stimulus” paradigm, where a brief, peripheral stimulus is presented while the infant is engaged with another stimulus, typically a video (Richards & Turner, 2001). In contrast to the “interrupted stimulus” paradigm, in the present study the interrupting stimulus is centrally presented and the infant does not have to make a gaze shift toward this stimulus; however, in both paradigms, the response evoked by the sudden‐onset checkerboard captures the trade‐off in infants’ attention distribution between the interrupting stimulus and the background video.

These neural measures allow us to adopt a principled approach and probe which of three hypotheses present in the literature best explains individual differences in visual sensory seeking in infancy. We first test whether visual sensory seeking differences reflect striving for *optimal stimulation*: in this case, we predict that lower visual seeking will associate with stronger VEPs (P1 peak amplitude) in response to the checkerboard (i.e., a measure of the strength of bottom‐up responsiveness to sensory input). We test the *processing speed* hypothesis by investigating the association between visual sensory seeking and the degree of change in frontal theta oscillatory amplitude with video repetition. In particular, we analyze the amplitude of the decrease in theta observed after repeatedly seeing the video and indexing the depletion of information. We predict that stronger decrease in theta amplitude, indexing faster processing of ongoing information, will associate with increased visual seeking. Finally, we test whether seeking relates to *information prioritization*—under this hypothesis, we expect higher visual seeking in those infants whose VEP responses (change in P1 peak amplitude) are stronger than expected based on their change in theta amplitude. Although we expect P1 and theta measures to inversely relate, individuals will depart from this regression line, with some exhibiting larger P1 changes than those expected from the decrease in theta and other participants exhibiting smaller changes. A larger than expected change captures a stronger bias given to incoming over ongoing information processing.

We quantify visual sensory seeking through the parent‐reported ITSP (Dunn, 2002). The sensory seeking quadrant of the ITSP in the visual modality provides a measure of infants’ active involvement in activities or actions such as looking at stimulating objects or attending to stimulating visual information (e.g., fast‐paced TV). Although elevated seeking of restricted and repetitive stimulation is reported in toddlers and children with ASD by studies using the ITSP or other age‐appropriate sensory questionnaires (SP and SSP; Dunn, 1999, 2014) (Ben‐Sasson et al., 2009; Lane et al., 2010; Liss et al., 2006; Simpson et al., 2019; Tomchek et al., 2018), decreased seeking is often documented in infants later developing ASD (Ben‐Sasson et al., 2007; Beranova et al., 2017; Mulligan & White, 2012). This evidence suggests that such sensory questionnaires may capture different constructs during early infancy as compared to later childhood. In particular, in early development, the ITSP visual sensory seeking items capture infants’ drive toward novel and diversive visual input, rather than restricted and repetitive stimulation. The developmental transition from early reduced sensory seeking to later elevated sensory seeking in ASD may reflect learning that one effective strategy infants later developing ASD have to limit incoming novel/diversive stimulation (i.e., which they may experience as distressing, Mulligan & White, 2012) is to seek restricted, repetitive and often self‐produced sensory stimulation.

Parents’ ability to detect and report on their infant's sensory behaviors is dependent on the child's developmental stage (Baranek, 1999; Stone & Hogan, 1993). Thus, some of the psychometric properties of the ITSP improve with the infant's developmental stage. For example, better internal consistency of the ITSP seeking quadrant is seen for the “7–36 months” version of the questionnaire, compared to the “0–6 months” version (Eeles et al., 2013). Therefore, we test these hypotheses in 10‐month‐old infants (and replicate significant results at 16 months). The second reason behind the choice of this age range lies in the qualitative shift in the nature of visual attention that occurs during the first year of life (Colombo, 2001; Johnson & De Haan, 2015). While infants aged 0–6 months tend to prioritize exogenously salient but simple visual stimuli, from 6 months infants’ attention begins to be drawn to more complex and naturalistic visual input (Reynolds & Romano, 2016). This is accompanied by a refinement of infants’ capacity to sustain attention to complex scenes, an ability that reaches functional maturity between 9 and 11 months (Colombo, 2001; Colombo & Cheatham, 2006). We, therefore, expect the 10 months age to be optimal to characterize the nature of individual differences in visual sensory seeking profiles through combination of parent‐reported and experimental measures.

## 2 METHODS

### 2.1 Participants

Forty‐eight healthy, full‐term 10‐month‐old infants (24 females, mean age = 10 months and 4 days, *SD* = 14 days) participated in the study. Five infants were tested, but not included in the final sample of participants because of low toleration of the EEG net, fussiness or excessive movement artifacts. Accordingly, 43 infants (22 females, mean age = 10 months and 4 days, *SD* = 14 days) were included in the final sample and contributed to the EEG and VEPs analyses. The minimum number of participants required was determined by an a priori power analysis (*Gpower*: Faul, Erdfelder, Lang, & Buchner, 2007; Faul, Erdfelder, Buchner, & Lang, 2009). According to Cohen (1988) and Sawilowsky (2009), a medium effect size in psychological studies is *r* = .50 and, considering an estimate power of .80, we estimate a sample size of 23 infants to detect one‐tailed correlational effects at an alpha‐level of 0.05 (24 infants to detect within‐group repeated‐measure effects at an alpha‐level of 0.05).

Infants were born full term (gestational age 38–42 weeks), weighed >2,500 g at birth, and had no history of pre or perinatal medical complications. All infants included in this research were typically developing and therefore had no known developmental atypicality, based on parental reports at recruitment. Participants were recruited from a volunteer database at the Centre for Brain and Cognitive Development (Birkbeck, University of London). Infants were tested if awake and in an alert state. The present study was conducted according to guidelines laid down in the Declaration of Helsinki, with written informed consent obtained from a parent or guardian for each infant before any assessment or data collection. All procedures involving human subjects were approved by the Research Ethics Committee at Birkbeck, University of London (Protocol no. 171805).

### 2.2 Stimuli

Experimental stimuli consisted of a background dynamic video clip selected from the animated cartoon *Fantasia* by Walt Disney and a black‐and‐white static checkerboard. The clip was 40 s long, it was repeated 10 times during the session and it was presented in the centre of the screen (covering a 22.5 cm wide × 12.5 cm vertical area, subtending a visual angle of 21° × 12°). The clip depicted dynamic, continuous, goal‐directed actions, accompanied by music. The black‐and‐white static checkerboard was presented for 100 ms, in the centre of the screen (covering a 30 cm wide × 30 cm vertical area, subtending a visual angle of 28° × 28°). The average luminance of the checkerboard was 1.56 cd/m^2^ for the black patch and 228 cd/m^2^ for the white patch. The checkerboard replaced the video clip which resumed following disappearance of the checkerboard from the interruption point.

### 2.3 Apparatus and procedure

Testing took place in a dimly illuminated room. Infants were seated on a parent's lap, 60 cm from a screen (27 inches; width: 59.77 cm, height: 33.62 cm). A two‐machine solution was adopted for experimental control. The sequence and timing of stimulus presentation was controlled using a computer with MATLAB^®^. This computer was interfaced with Net Station (Electrical Geodesic) via a serial connection. Net Station was used to record the sequence of events along with the high‐density EEG data. Continuous scalp EEG was recorded from 124 channels of a 128‐channel HydroCel Geodesic Sensor Net that was connected to a NetAmps 400 amplifier (Electrical Geodesic) and referenced online to the vertex (Cz). Channel impedance was kept at or below 100 KΩ, and signals were sampled at 500 Hz. A video camera situated below the screen used for stimulus presentation recorded the infants’ face and gaze behavior. This information was used for online monitoring of infants’ performance. Further, infants’ videos were saved and stored for offline behavioral coding.

**FIGURE 1:**
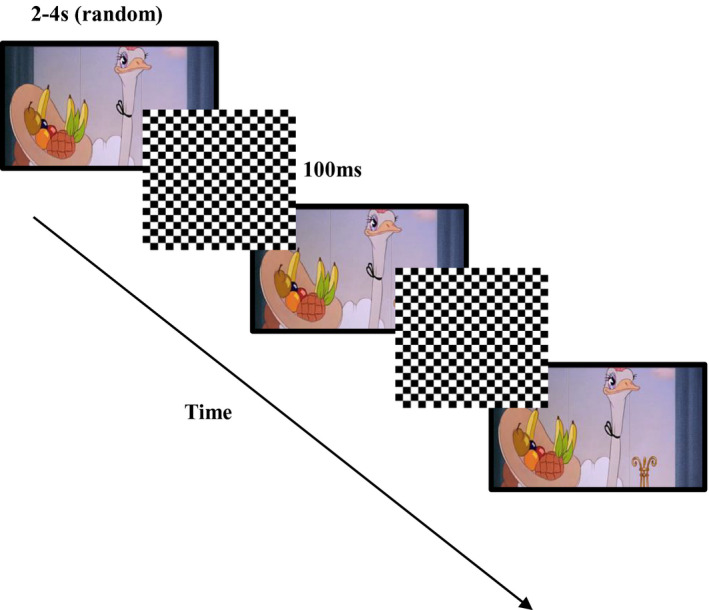
Representation of the sequence of events in the experimental paradigm. A 40‐s‐long video clip from the animated cartoon *Fantasia* was presented accompanied by music 10 times and randomly interrupted by the appearance of 128 black‐and‐white static checkerboards (100 ms) flashed on top (ISI = 2–4 s, random)

As shown in Figure 1, each trial began with the presentation of the video clip accompanied by music. Music was used throughout the task to promote infants’ engagement with the visual scene. Further, visual and auditory stimuli remained synchronous throughout the task. The same clip was repeated 10 times during the session and intermixed with presentation of 128 black‐and‐white static checkerboards flashed on top (ISI = 2–4 s, random). The time points (within the background video) when this stimulus was presented were the same for all participants. A photodiode connected to an oscilloscope was used to measure the onset of checkerboards. Music was not paused during checkerboard presentation since this stimulus lasted only 100 ms.

The total experimental session duration was 8 min but the experimenter could interrupt the experiment earlier, in case of infant's fussiness, prolonged inattention or if requested by the parent. No behavioral criterion of cognitive habituation was employed (e.g., looking time). Rather, frontal theta oscillatory amplitude provided a direct measure of infants’ progressive engagement and disengagement with the ongoing repeated video clip (Xie, Mallin, & Richards, 2019).

### 2.4 Infant‐Toddler sensory profile

At experiment completion, caregivers were asked to fill in the parent‐reported questionnaire ITSP (Dunn, 2002). Further, parents were re‐contacted six months after their infant participated to fill in the ITSP online. The “7–36 months” version of the ITSP is a 48‐item questionnaire which provides a measure of infants’ sensory processing in four quadrants (i.e., sensory seeking, low registration, sensation avoiding and sensory sensitivity) for each sensory domain. Visual sensory seeking is captured through four items asking whether the child enjoys looking at moving or spinning objects (Item 14); enjoys looking at shiny objects (Item 15); enjoys looking at own reflection in the mirror (Item 19); and prefers fast‐paced, brightly colored TV shows (Item 20). Parents were asked to rate the frequency of occurrence of infant's sensory behaviors on a 5‐point scale (i.e., 1 = almost always; 5 = almost never). In a normative sample, the reliability of the domains and quadrants’ scores ranges from 0.69 to 0.85 (Dunn, 2002; Eeles et al., 2013), and good content and criterion validity are reported (Dunn & Daniels, 2002). In order to test the hypothesis that parent‐reported individual sensory seeking profiles in the visual domain associate with infants’ ability to modulate responsiveness to incoming sensory input in our task, infants’ average scores for the sensory seeking quadrant in the visual domain were extracted and included in the subsequent statistical analyses at both time points. While excellent internal consistency (Cronbach's α = 0.86) is reported for the seeking quadrant of the “7–36 months” version of the ITSP (Eeles et al., 2013), it is worth noting that extracting only few items from the questionnaire may undermine construct validity and reliability. Thus, we report in Appendix S1 internal consistency and composite reliability for the four visual sensory seeking items extracted from the ITSP at 10 and 16 months. Further, we report internal consistency for the overall sensory seeking quadrant at both age points.

### 2.5 Infants’ gaze behavior coding

Infants’ gaze behavior was coded offline with a computerized frame‐by‐frame observational coding system (25 frames/s—Mangold, 2010), enabling two independent coders to identify screen‐directed looking (coded as 1) and looking away (coded as 0). Offline coding was used for the purpose of EEG data processing and analysis. Trials in which the infant did not look at the screen from 1 s before checkerboard onset until 1 s after checkerboard offset were excluded from the analysis. To ascertain reliability, the second observer independently coded a random 30% of video files (i.e., 13 participants). An inter‐rater reliability analysis using Cohen's Kappa was performed on the coded individual trials to determine consistency among observers. This analysis indicated that there was high agreement among the observers, κ = 0.992, (95% CI, 0.983 to 0.997), *p* < .001.

### 2.6 EEG recording and analysis

EEG data were pre‐processed offline using Net Station (Electrical Geodesic). Continuous EEG data were filtered using a 0.3–40 Hz band‐pass filter. As a first step, the EEG signal was segmented from 500 ms prior to checkerboard onset through 1,500 ms after checkerboard onset. Automated artifact detection was applied to the segmented data to detect individual epochs that showed >200 μV voltage changes within the segment period. EEG recordings were manually inspected and individual channels within segments were eliminated from the analysis if artifacts occurred. Segments whereby infants did not look at the screen as indicated by behavioral coding were further excluded from analysis. Segments in which >15% of the electrode channels were marked as bad were excluded from the analysis. For the remaining trials, spherical spline interpolation was conducted to replace data for bad channels using the five closest electrodes. Infants were excluded from the analysis if they had less than 10 artifact‐free segments (*n* = 2, included in the total count of 5 infants excluded from the study). Artifact‐free data were binned into four consecutive time intervals, each consisting of maximum 32 segments. Binning of artifact‐free data was implemented to estimate a measure of intra‐participant modulation of VEPs time‐locked to checkerboard presentation and EEG frontal theta time‐locked to video clip presentation. The choice of four time bins was made to achieve optimal balance between (a) having enough trials per time bin to maximize the signal‐to‐noise ratio and (b) having enough time bins to estimate non‐linear modulatory effects in the extracted EEG measures. On average, the mean number of segments by which infants contributed to the analysis of VEPs time‐locked to checkerboard presentation and EEG frontal theta during video clip presentation was *M* = 30.74, *SD* = 4.12 for bin 1, *M* = 29.13, *SD* = 6.54 for bin 2, *M* = 25.69, *SD* = 8.29 for bin 3, and *M* = 22.46, *SD* = 8.23 for bin 4. Results of statistical analyses are reported below with and without inclusion of number of valid trials as covariate.

#### 2.6.1 Visual‐evoked potentials (VEPs)

**FIGURE 2:**
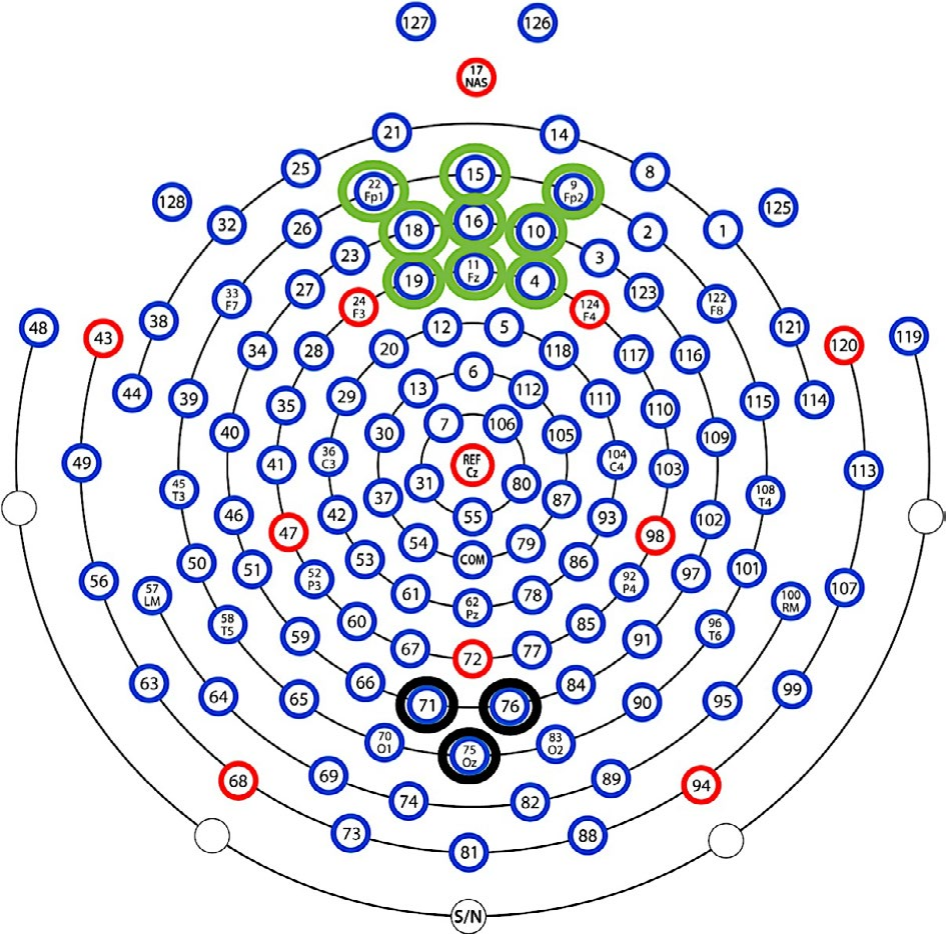
Hydrocel Geodesic Sensor Net montage displaying the occipital (black circle) and frontal (green circle) clusters of electrodes used for quantifying, respectively, VEPs time‐locked to checkerboard onset and theta amplitude during video presentation

**FIGURE 3:**
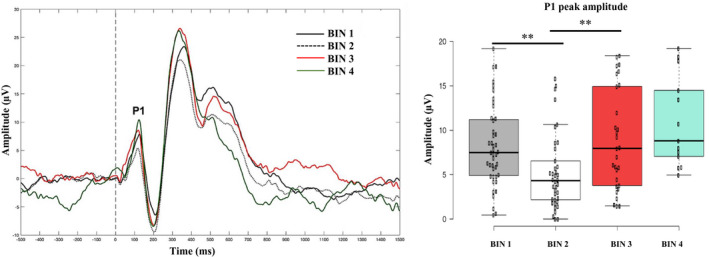
Grand‐averaged VEP response time‐locked to checkerboard onset for each time bin (gray solid line = bin 1; gray dotted line = bin 2; red solid line = bin 3; green solid line = bin 4). Boxplots illustrate the non‐linear modulation of the P1 peak amplitude, which significantly decreased from bin 1 to bin 2 and significantly increased from bin 2 to bin 3 (gray = bin 1; white = bin 2; red = bin 3; green = bin 4). **p < .001

To quantify VEPs time‐locked to checkerboard onset, averaged waveforms were generated for each of the extracted bins, re‐referenced to average reference and baseline corrected by subtracting the average of the 100 ms pre‐stimulus period. Inspection of the grand‐averaged waveform revealed that the P1 component was reliably elicited at checkerboard onset over the occipital scalp site. Based on previous literature (Richards, 2000) and on visual inspection of both the grand‐averaged and individual waveforms, channels (CH) 71, 75, and 76 (see Figure 2) were clustered and the average activity over these channels was computed for each participant, and each of the four bins. Based on the individual and grand‐averaged data, as well as on previous literature (Richards, 2000; Xie & Richards, 2017), the peak amplitude of the P1 was extracted within a time window of 100–150 ms following checkerboard onset (see Figure 3).

#### 2.6.2 EEG frontal theta oscillatory amplitude (4–6 Hz)

**FIGURE 4:**
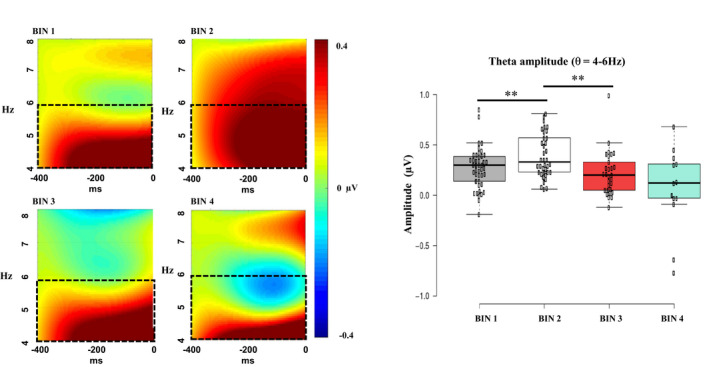
Grand‐averaged frontal theta amplitude during video clip for each time bin. Dotted squares indicate the 4–6 Hz frequency range of interest. Amplitude scale is −0.4, 0.4 µV for each time bin. Boxplots illustrate the non‐linear modulation of frontal theta amplitude, which significantly increased from bin 1 to bin 2 and significantly decreased from bin 2 to bin 3 (gray = bin 1; white = bin 2; red = bin 3; green = bin 4). **p < .001

To quantify EEG frontal theta oscillatory amplitude time‐locked to video clip presentation, segments for each of the extracted bins were subjected to time‐frequency decomposition. Artifact‐free segments were imported into MATLAB^®^ using the free toolbox EEGLAB (v. 13.4.3b) and re‐referenced to average reference. The collection of scripts *WTools* (developed by E. Parise, L. Filippin, & G. Csibra, available upon request) was used for spectral decomposition, employing complex Morlet wavelets for the frequencies 3–20 Hz (1 Hz resolution; real‐valued Gaussian with 3.5 cycles per time unit). Total induced oscillations were computed by performing a continuous wavelet transformation of all segments by means of convolution with each wavelet and by taking the absolute value of the results. To remove the distortion introduced by convolution at segment ends, 1,000 ms zero‐padding was performed and segments were chopped to obtain epochs indexing the activity occurring during a 400 ms‐long period of video clip presentation before checkerboard onset. The epochs were averaged for each time bin. Inspection of the time‐frequency plots revealed that 4–6 Hz frontal theta was reliably elicited in response to the video clip over the frontal scalp site. Previous research indicates that phases of information encoding are accompanied by a sharp increase in 4–6 Hz frontal theta in infants aged 10–11 months (Begus et al., 2015; Orekhova et al., 2006). Based on previous literature and on visual inspection of both the grand‐averaged and individual time‐frequency plots, channels (CH) 4, 9, 10, 11, 15, 16, 18, 19, and 22 (see Figure 2) were clustered and the average 4–6 Hz activity extracted during the 400 ms of video clip presentation before checkerboard onset for each of the four time bins (see Figure 4; Table 1).

**TABLE 1:** Mean and standard error of the P1 peak amplitude and 4–6 Hz frontal theta amplitude for each of the four time bins

Mean (*SE*)	BIN 1	BIN2	BIN3	BIN 4
P1 peak amplitude	8.22 (0.694)	5.21 (0.679)	8.86 (1.04)	10.91 (1.36)
Theta amplitude	0.270 (0.030)	0.404 (0.039)	0.219 (0.040)	0.062 (0.108)

## 3 EXPERIMENTAL PREDICTIONS

### 3.1 Trade‐off in infants’ visual attention distribution

The paradigm was designed to capture a trade‐off in infants’ visual attention distribution to the background repeated video clip and checkerboard stimuli. We predicted frontal theta oscillatory amplitude to the repeated video to manifest initial sustained synchronization, followed by later desynchronization. Conversely, we expected the P1 peak amplitude to be inversely modulated, exhibiting an increase as theta decreases with repetition. We further predicted the two measures to be negatively correlated throughout the experimental session.

### 3.2 Source of individual differences in visual sensory seeking

Under the *optimal stimulation hypothesis*, we predicted infants with lower overall P1 peak amplitude to be rated as “high visual seekers”; conversely, we predicted infants with higher overall P1 peak amplitude to be rated as “low visual seekers.” Under the *processing speed hypothesis*, we predicted infants manifesting faster decline in frontal theta amplitude after repeatedly seeing the video to afford seeking more information, thus being rated as “high visual seekers”; conversely, infants manifesting slower decline in frontal theta amplitude should afford less, thus being rated as “low visual seekers.” Finally, under the *information prioritization hypothesis*, we expected infants exhibiting a modulation of the P1 peak amplitude stronger than expected based on the change in theta amplitude (i.e., more weight allocated to incoming over ongoing information processing) to be rated as “high visual seekers”; conversely, we predicted infants exhibiting a modulation of the P1 peak amplitude weaker than expected based on their change in theta amplitude (i.e., less weight allocated to ongoing over incoming information processing) to be rated as “low visual seekers.”

## 4 RESULTS

### 4.1 Change in theta oscillatory amplitude and P1 peak amplitude during the task

As a first step, a Generalized Estimated Equation approach assuming a Gaussian distribution and identity link was used to investigate the modulation of the peak amplitude of the P1 time‐locked to checkerboard onset and frontal theta amplitude time‐locked to video clip presentation as a function of time (bin). This method was chosen to account for within‐subject correlations and to handle missing data consequent to not all infants completing the experimental session. Wald tests were computed to determine the significance of the effects in both cases.

For frontal theta amplitude, a significant main effect of bin was observed (Wald*χ* ^2^(3) = 23.22, *p* < .001). This result did not change when the number of valid trials for the four time bins was added as a covariate (Wald*χ* ^2^(3) = 21.94, *p* < .001). Bonferroni‐corrected pairwise comparisons indicated that frontal theta amplitude significantly increased from bin 1 to bin 2 (*p* < .001), significantly decreased from bin 2 to bin 3 (*p* < .001), and did not change from bin 3 to bin 4 (*p* = .128). For the peak amplitude of the P1, a significant main effect of bin was observed (Wald*χ* ^2^(3) = 53.69, *p* < .001). This result did not change when number of valid trials for the four time bins was added as a covariate (Wald*χ* ^2^(3) = 55.21, *p* < .001). Bonferroni‐corrected pairwise comparisons indicated that the peak amplitude of the P1 significantly decreased from bin 1 to bin 2 (*p* < .001) and significantly increased from bin 2 to bin 3 (*p* < .001). No change was observed from bin 3 to bin 4 (*p* = .115).

### 4.2 Association between P1 peak amplitude and theta oscillatory amplitude

A repeated measure correlation was run to assess the association between the P1 peak amplitude and frontal theta amplitude for the four time bins. This statistical approach was chosen to account for the non‐independence of observations and preserve individual differences present in the data. The package “*rmcorr*” was used (Bakdash & Marusich, 2017; R Core Team, 2017). This test was statistically significant (*r_rm_*(79) = −.25, *p* = .025, 95% CI [−0.45, −0.029]), indicating that the higher the engagement with the video stimulus, as indexed by frontal theta oscillatory amplitude, the lower the responsivity to the checkerboard, as indexed by the peak amplitude of the P1. Additionally, the negative association between P1 peak amplitude and frontal theta amplitude held within each of the four time bins. This result confirmed the capability of the paradigm to capture a trade‐off in infants’ visual attention distribution to the background video and checkerboard stimuli.

**FIGURE 5:**
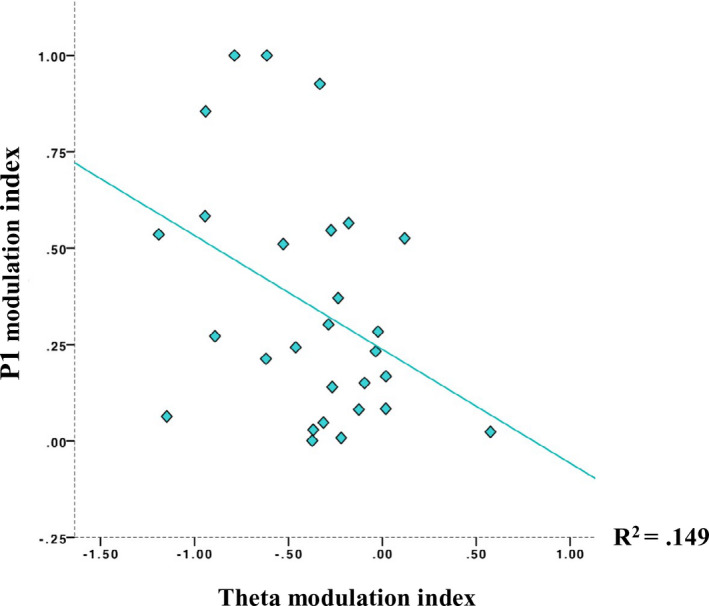
Scatter plot illustrating the association between theta modulation index: [theta bin 3 − theta bin 2]/[theta bin 3 + theta bin 2] and P1 modulation index: [P1 bin 3 − P1 bin 2]/ [P1 bin 3 + P1 bin 2]. The stronger the modulation of frontal theta amplitude to the video, the stronger the modulation of the P1 peak amplitude to the checkerboard. Above the regression line are infants whose P1 change is larger than expected from theta change, below the regression line are infants whose P1 change is smaller than expected from theta change

To further characterize the dependency between theta and P1, the scaled difference in frontal theta amplitude and in the peak amplitude of the P1, respectively, were computed between bin 3 and bin 2 for each infant (i.e., theta modulation index: [theta bin 3 − theta bin 2]/[theta bin 3 + theta bin 2]; P1 modulation index: [P1 bin 3 − P1 bin 2]/[P1 bin 3 + P1 bin 2]). These time bins were chosen for three reasons: (a) they suffered less form data loss than bin 4 did (29 participants with 3 bins, 13 with 4 bins), (b) the change between bins 2 and 3 was on average larger than between bins 1 and 2, thus providing more variance for the analysis, and (c) conceptually, the decrease in theta amplitude (rather than the increase occurring from bin 1 to 2) was closer to a measure of information depletion (Clarke et al., 2018). An individual data point deviating more than 2 *SD* from the mean was removed prior to the analysis. Normality assumptions were assessed and no violation was detected. A Pearson correlation yielded a statistically significant association (*r*(27) = −.386 *p* = .021, *R* ^2^ = .149), indicating that the stronger the modulation of frontal theta amplitude, the stronger the modulation of the P1 peak amplitude (see Figure 5). This result confirmed evidence from the repeated measure correlation analysis. An additional analysis on the change between bin 1 and bin 2 was run and results are reported in Appendix S1.

### 4.3 Association with visual sensory seeking

In order to investigate the source of individual differences in parent‐reported visual sensory seeking, infants’ average scores for the sensory seeking quadrant in the visual domain were first computed. We investigated the associations between this measure and (a) the overall P1 peak amplitude (taken as a measure of the strength of bottom‐up responsiveness to sensory input), (b) the change in frontal theta oscillatory amplitude from bin 2 to 3 (indexing the speed of information processing), and (c) the degree of modulation of the P1 peak amplitude by ongoing theta amplitude (taken as a measure of how successful incoming sensory input was in capturing infants’ attention away from the ongoing video infants were engaged with).

**FIGURE 6:**
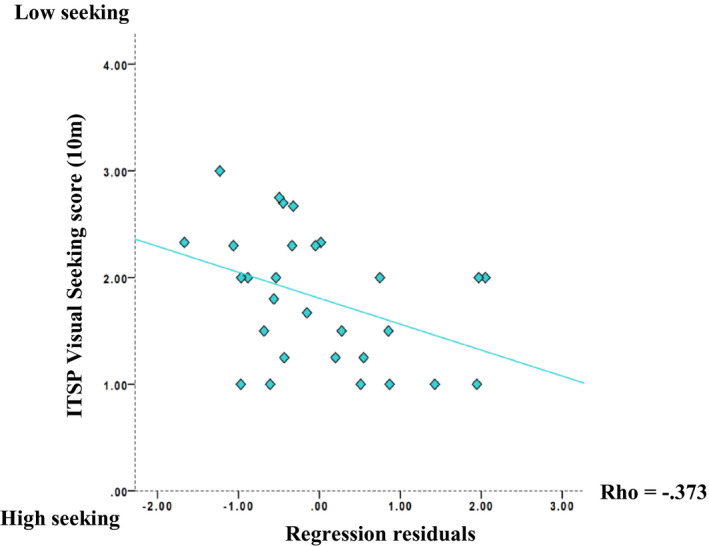
Scatter plot illustrating the association between the ITSP visual seeking scores at 10 months and the residuals of a regression with the theta modulation index as predictor and the P1 modulation index as outcome. Infants whose P1 modulation index was higher than predicted by theta amplitude change were rated as “high visual seekers.” Infants whose P1 modulation index was lower than predicted by theta amplitude change were rated as “low visual seekers.” Note that the range for the y axis starts at zero for ease of visualization

Since the distribution of the visual sensory seeking variable violated the normality assumptions (Shapiro–Wilk test, *p* = .034), a bivariate Spearman correlation was run to assess the relationship between this measure and the overall P1 peak amplitude. This test was not statistically significant (*r*_s_(41) = −.065, *p* = .681). Infants visual seeking scores were similarly not related to modulation of ongoing theta (i.e., theta modulation index), (*r*_s_(27) = −.067, *p* = .728). Rather, they significantly associated with the degree of peak amplitude modulation of the P1 component (i.e., P1 modulation index), (*r*_s_(27) = −.359, *p* = .028). These contrasting results indicate that there was individual variation in the degree to which theta changes modulated change in the P1 peak amplitude. In order to directly assess whether this source of variation explained individual differences in visual sensory seeking profiles, we extracted residuals from a linear regression having the theta modulation index as predictor and the P1 modulation index as outcome. A bivariate Spearman correlation between the infants’ visual sensory seeking scores and the regression residuals was computed. This test was statistically significant, (*r*_s_(27) = −.373, *p* = .023). The negative direction of this correlation indicated that those infants who exhibited a modulation of the P1 peak amplitude greater than that predicted by change in frontal theta amplitude, that is, a stronger increase in P1 peak amplitude, were rated by parents as “high visual seekers.” Conversely, infants who exhibited a reduced modulation of the P1 peak amplitude than that predicted by change in frontal theta amplitude were rated by parents as “low visual seekers” (see Figure 6). In both cases, the item most strongly correlating with these measures was item 20, which asks whether the child prefers fast‐paced, brightly colored TV shows (see Appendix S1).

Overall, these results confirmed the hypothesis that variation in infants’ seeking of visual stimulation reflects individual differences in orienting away from ongoing to incoming stimulation. At the same level of information uptake from the ongoing video, high seekers were already disengaging with it and ready to engage with incoming stimulation, while low seekers remained engaged with the ongoing video. In the Appendix S1, we report that these associations remained significant when the follow‐up ITSP collected at 16 months was used in the analysis. Further, these associations appear to be specific to the visual domain since neither P1 modulation index, nor the regression residuals, significantly associated with the ITSP total sensory seeking scores (which include visual, auditory, tactile, oral, and vestibular seeking). Finally, an additional analysis in Appendix S1 makes it unlikely that our findings are explained by early TV exposure in our participants, thus reinforcing our hypothesis that it is infants’ information processing bias that explains their concurrent and later visual sensory seeking profiles.

### 4.4 Relative explanatory power of the three hypotheses

Results from previous analyses did not support either the optimal stimulation hypothesis or the processing speed hypothesis as potential explanations for individual differences in infant visual sensory seeking. However, the absence of evidence does not allow to conclude that these hypotheses carry no explanatory power for the current dataset. Thus, a hierarchical linear regression with 10‐month visual sensory seeking as outcome and each of the predictors entered to the model at separate steps was performed.

First, the P1 modulation index was entered to the model as predictor. The model was statistically significant, *F*(1, 27) = 4.068, *p* = .027, $R_{\text{adj}}^{2}$ = .131, confirming the explanatory power of the information prioritization hypothesis. In step 2, the theta modulation index was entered to the model as a predictor. The model was no longer statistically significant, *F*(2, 25) = 1.976, *p* = .160, $R_{\text{adj}}^{2}$ = .067, and did not account for a higher proportion of variance relative to a model with the only P1 modulation index as predictor, *F* change (1, 25) = 0.609, *p =* .442. In step 3, the overall P1 peak amplitude was added to the model as predictor. The model was not statistically significant, *F*(3, 24) = 1.976, *p* = 1.354, $R_{\text{adj}}^{2}$ = .038, and did not account for a significantly higher proportion of variance relative to a model with the only P1 modulation index as predictor, *F* change (1, 24) = .231, *p =* .635. These results indicated that neither the processing speed hypothesis, nor the optimal stimulation hypothesis added additional explanatory power for the current dataset.

## 5 DISCUSSION

The goal of the present study was to adopt a principled approach to explain infants’ individual differences in visual sensory seeking, defined in our study as differences in infants’ seeking of novel visual stimulation. 10‐month‐old infants saw 10 repetitions of a video clip briefly interrupted by black‐and‐white static checkerboards overlaid on top. EEG/VEP responses were recorded. Separate indices of infants’ information processing progress (i.e., modulations of frontal theta oscillatory amplitude to the video) and stimulus selection (i.e., modulations of the P1 peak amplitude to the sudden‐onset checkerboard) during the task were extracted and related to parental reports of infants’ sensory seeking in the visual modality (measured by the ITSP at 10 and 16 months).

First, we demonstrated the capability of the paradigm to capture a trade‐off in infants’ attention distribution to the background video and flashed checkerboard stimuli. Frontal theta oscillatory amplitude to the repeated presentation of the video clip manifested a non‐linear modulatory profile, which reflected the progressive encoding and depletion of information (Nordt et al., 2016). Although we initially hypothesized theta oscillatory amplitude to manifest a profile of initial sustained activation, followed by a decrease, we actually observed an increase from bin 1 to bin 2. Other studies have characterized an initial phase of gradual increased engagement with information. For example, infants become less distractible as a look toward a video stimulus progresses (Richards & Turner, 2001). The mechanism involved remains unknown but some have observed changes in scanning from shorter to longer fixations made to adjacent regions of the scene, as adult participants viewed video material (Fischer, Graupner, Velichkovsky, & Pannasch, 2013; Pannasch, Helmert, Roth, Herbold, & Walter, 2008). While this explanation remains speculative, it is possible that, when presented with new information (i.e., the unfamiliar video clip), infants initially explored the scene before fully engaging with its contents to extract information about particular aspects of the video. More importantly for the hypotheses under investigation, the P1 peak amplitude to sudden‐onset checkerboards was non‐linearly modulated and exhibited a profile that was inversely related to theta oscillatory amplitude. The paradigm was, therefore, deemed optimal to test three hypotheses proposed to explain individual differences in seeking novel stimulation in the visual modality.

We first tested the *optimal stimulation hypothesis*, according to which individuals’ active engagement with their environment strives to achieve an optimal level of stimulation (Zentall & Zentall, 1983). According to this hypothesis, individuals seek stimulation if they are under‐responsive to current sensory input. Under this hypothesis, we predicted that higher parent‐reported visual seeking would associate with weaker VEPs (i.e., overall P1 peak amplitude) in response to incoming stimulation (i.e., checkerboards). We found no evidence in support of this hypothesis. Infants rated by parents as high visual seekers did not exhibit reduced P1 peak amplitude in our task. The *optimal stimulation hypothesis* draws heavily on research with atypical populations and evidence supporting this account is scarce in neurotypical individuals (Carrol, Zuckerman, & Vogel, 1982). It is possible that only under conditions of extreme sensory input (e.g., sensory overload or deprivation) would typically developing individuals make use of compensatory strategies resembling those observed in atypical populations. Further, while some evidence for the optimal stimulation hypothesis exists in older children with ASD (Donkers et al., 2015), our study is the first to assess this hypothesis in infancy.

Second, we tested the *processing speed hypothesis*, according to which individual differences in seeking novel stimulation reflect differences in information processing abilities (Colombo et al., 1991). Under this hypothesis, we predicted that higher visual seeking would associate with more rapid information processing, as indexed by a stronger decrease in frontal theta amplitude with repetition of the video in our task. Our results did not support this hypothesis either. Infants’ modulation of EEG frontal theta to the video was not related to parent‐reported visual seeking profiles. Information processing progress was proposed as a potential driver of attention and sensory seeking (Gottlieb et al., 2013); however, our findings suggest that infants speed of processing information is insufficient to account for individual differences in visual sensory seeking profiles.

From early in development, infants are equipped with the ability to actively acquire information and modulate their learning on the basis of their own exploratory drives (Begus, Gliga, & Southgate, 2014; Begus & Southgate, 2018). Thus, individual biases in *information prioritization* might associate with alternative seeking profiles. Under this hypothesis, we predicted higher visual seeking in those infants whose VEP responses (i.e., P1 peak amplitude) were stronger than expected based on their theta amplitude, thus attributing stronger weight to incoming relative to ongoing information processing. Evidence from our study confirmed this hypothesis. Infants rated as “high visual seekers” exhibited an increase in P1 peak amplitude from bin 2 to 3 greater than that predicted by the concurrent decrease in frontal theta oscillatory amplitude (with the opposite occurring in infants rated as “low visual seekers”). This result suggests that a bias toward incoming stimulation characterized the sensory behavior of high seeking infants. At the same degree of information uptake, high seeking infants (but not low seeking infants) were more readily disengaging from ongoing stimulation to orient to incoming input.

Our study made use of the ITSP to capture parent‐reported visual sensory seeking profiles at 10 and 16 months. Interestingly, among the four items contributing to the ITSP visual sensory seeking score, the item that at both time points explained the highest proportion of variance in the EEG measures was item 20, which asks if the child prefers fast‐paced, brightly colored TV shows. This item maximally captures infants seeking of novel visual stimulation. Further, the strength of the association between item 20 and the EEG measures increased from the 10 to 16 months—a result which might be consequent to the larger sample size (i.e., fewer parents rated this question as non‐applicable and thus the sample answering this question was larger at 16 months). Having replicated the associations with the ITSP at 16 months also gives us confidence that we are capturing a stable and reliably reported trait.

The associations between task performance and infants’ seeking found in our study were specific to the visual modality. Individual differences in disengaging from ongoing stimulation to orient to incoming input did not associate with infants’ seeking scores averaged across modalities. One reason behind this result might be the poor reliability of the seeking quadrant observed for some of the ITSP sensory modalities (i.e., Cronbach's α at 10 months [auditory] = 0.231; [tactile] = 0.439; [vestibular] = 0.450; at 16 months [auditory] = 0.465; [tactile] = 0.680; [vestibular] = 0.587). Further, our experimental paradigm was designed to capture a trade‐off in the allocation of attentional resources in the visual modality. Therefore, it comes to no surprise that task‐related differences were only explaining alternative visual seeking profiles. However, we expect similar principles to apply to other sensory modalities (Frost, Armstrong, Siegelman, & Christiansen, 2015). Future studies should capitalize on our task and apply adapted versions to the investigation of the auditory or tactile modalities.

Although we test the extent to which data supported three hypotheses, we do not conceptualize these hypotheses as being necessarily mutually exclusive. For example, it is possible that different mechanisms may best explain sensory seeking profiles in typical versus atypical development (e.g., infants at elevated likelihood of ASD or alternative atypical developmental outcome; Williams et al., 2018). Further, it is possible that a combination of these hypotheses (e.g., optimal stimulation and information prioritization hypotheses) may better account for individual differences in sensory seeking in infants with later atypical development.

Visual orienting to incoming sensory events is known to enhance neural responses in primary visual areas (Ranganath & Rainer, 2003), and this orienting response is influenced by dopamine receptor polymorphisms (Lakatos et al., 2003). Influences of these polymorphisms have been reported on neonatal and infant temperament (Ebstein et al., 1998; Lakatos et al., 2003), as well as adult personality traits (Benjamin et al., 1996; Ebstein et al., 1996). For example, the dopamine‐transporter gene DRD4 exists in two common forms, the 4‐repeat variety and the 7‐repeat form. The 7‐repeat variety of DRD4 is less sensitive to dopaminergic influences than the 4‐repeat form and infants as young as 12 months with this transporter gene are reported to be less anxious and driven toward novelty than those with the shorter version. Further, the DRD4 7‐repeat form has been associated with conditions characterized by extreme sensory seeking behaviors such as ADHD (Comings et al., 1999; Swanson et al., 1998). Thus, our experimental paradigm might offer an intermediate phenotype between genes and behavior that will help better characterizing both typical and atypical sensory seeking.

Another question is to what extent the drive toward novel stimulation which we capture with our measures maps onto higher levels of information seeking manifested later in development through pointing (Begus & Southgate, 2012) or questioning (Kurkul & Corriveau, 2018). Indeed, a distinction is made in adult self‐report questionnaires between seeking perceptual as opposed to epistemic novelty, the former inquiring, for example, about the need to take a closer look at something perceived in the distance and the latter covering manifestations like the need to solve problems or the enjoyment of learning something new (Litman & Spielberger, 2003; Piotrowski, Litman, & Valkenburg, 2014). This is an important question awaiting future empirical investigation. We speculate that our measure of prioritization of information will capture stable individual differences with variable behavioral manifestations as children discover new means to actively seek or elicit new information.

The trade‐off between information processing progress (indexed by frontal theta oscillatory amplitude modulation) and bias toward incoming stimulation (indexed by P1 peak amplitude modulation) highlighted by this research supports developmental theories portraying *optimal learning* as evidenced by a shift from exploitation of the resource at hand to exploration of incoming sensory input (e.g., Cohen et al., 2007; Mather, 2013; Twomey & Westermann, 2018). The specificity of our paradigm lies in its ability to characterize these interacting mechanisms at a neural level. Moreover, our study suggests that deviations from *optimal learning* may manifest early in development. We show, for the first time, that individual differences in the prioritization of incoming relative to ongoing stimulation can potentially explain parent‐reported sensory profile differences emerging toward the end of the first year of life. We speculate that preserving individual variation in how we assign relative value to ongoing relative to incoming stimulation and in how we are differentially drawn to seek sensory input carries an evolutionary advantage, in that it promotes discovery, at a population level, contemporarily fostering learning and consolidation of acquired knowledge.

## Supporting information

Appendix S1
