# Supplementary material for: Explaining individual differences in infant visual sensory seeking
Source: Infancy. 2020 Aug 3;25(5):677–98. doi: 10.1111/infa.12356 (PMC7496506; doi:10.1111/infa.12356)
Supplement: Supplementary file 1 — Appendix S1 [file INFA-25-677-s001.docx]

**Supplementary materials**

**Explaining individual differences in infant visual sensory seeking**

Elena Serena Piccardi^1^, Mark H. Johnson^1,2^, Teodora Gliga^1,3^

(1) Centre for Brain and Cognitive Development, Birkbeck University of London, London (UK)

(2) Cambridge University, Department of Psychology, Cambridge (UK)

(3) University of East Anglia, Department of Psychology, Norwich (UK)

Corresponding author: Elena Serena Piccardi

Address: Centre for Brain and Cognitive Development, Malet Street London, WC1E 7HX.

E-mail address: [epicca01@mail.bbk.ac.uk](mailto:epicca01@mail.bbk.ac.uk)

**Running title:** Individual differences in infant visual sensory seeking

**Acknowledgements**

The authors would like to thank all the families who participated in the study. This work was supported by MRC Programme Grant no. G0701484. The authors declare no conflicts of interest with regard to the funding source for this study.

**Methods**

*Infant-Toddler sensory profile (ITSP) reliability*

First, we investigated reliability of the ITSP sensory seeking quadrant at 10 and 16 months by extracting Cronbach’s α. At 10 months, Cronbach’s α = 0.811; at 16 months, Cronbach’s α = 0.774, thus indicating satisfactory internal consistency. Second, we assessed reliability of the ITSP sensory seeking items in the visual modality at both age points. Since Cronbach’s α depends on the number of items and tends to underestimate internal consistency with fewer items (Tavakol & Dennick, 2011), we also extracted composite reliability at both age points. At 10 months, Cronbach’s α = 0.561 and CR = 0.751, indicating satisfactory internal consistency. At 16 months, Cronbach’s α = 0.521 and CR = 0.663, confirming acceptable internal consistency.

**Results**

**Additional analyses**

*Association with overall sensory seeking scores*

To assess the domain specificity of the associations reported in the manuscript, we extracted an overall sensory seeking score by averaging across sensory modalities. We investigated the associations between this measure and (1) the overall P1 peak amplitude, (2) the change in theta amplitude and (3) the degree of modulation of the P1 component by ongoing theta amplitude. Normality assumptions were assessed and no violation was detected. A bivariate Pearson correlation between the P1 peak amplitude and the overall sensory seeking scores at 10 months yielded an insignificant association between the measures, *r* (41) =-.019, *p* = .904, *R^2^* = .0003. Infants sensory seeking scores were similarly not related to modulation of ongoing theta amplitude (i.e., theta modulation index), *r* (27) = -.229, *p* = .240, *R^2^* = .053 or to the degree of peak amplitude modulation of the P1 component by ongoing theta amplitude (i.e., P1 modulation index), *r* (27) = -.032, *p* = .872, *R^2^* = .001. Following the same analytical pipeline reported in the manuscript, we extracted residuals of a regression with the theta modulation index as predictor and P1 modulation index as outcome. The bivariate Pearson correlation between the regression residuals and the overall sensory seeking scores was not statistically significant, *r* (27) = -.088, *p* = .649, *R^2^* = .008. Overall, this additional analysis confirmed the modality specificity of the effects reported in the manuscript.

*Analysis on bin 1 and bin 2*

To further support results of the analyses run on bin 2 and bin 3, the same analytical pipeline was conducted on bin 1 and bin 2. First, a theta modulation index and a P1 modulation index were quantified for bin 1 and bin 2 using the procedure reported in the manuscript. Normality assumptions for the two indexes were assessed and no violation was detected. A Pearson correlation between the theta modulation index and the P1 modulation index yielded a statistically significant association (*r* (36) = .288, *p* = .040, *R^2^* = .083), indicating that the stronger the modulation of EEG frontal theta amplitude, the stronger the modulation of the P1 peak amplitude.

Since the distribution of the visual sensory seeking variable violated normality assumptions (Shapiro-Wilk test, *p* = .034), a bivariate Spearman correlation was run to assess the association between the visual seeking scores and the theta modulation index. The result was not statistically significant, (*r_s_* (36) = -.013, *p* = .940). The association between the P1 modulation index and the visual seeking scores was also insignificant (*r_s_* (36) = .080, *p* = .316), but the direction of the relationship was the same found for bin 2 / bin 3 and reported in the manuscript. We extracted residuals of a regression having the theta modulation index as predictor and the P1 modulation index as outcome. A bivariate Spearman correlation between the infants’ visual sensory seeking scores and the regression residuals was computed. This test was not statistically significant (*r_s_* (36) = .105, *p* = .265). Despite lacking statistical significance, the direction of this association was the same found for later time bins, i.e. infants who maintained a strong P1 peak amplitude despite the concurrent increase in frontal theta amplitude were rated by parents as ‘high visual seekers’.

Since a moderate change in engagement with the video stimulus occurred during the first two time bins (theta modulation index SD=.34) relative to later time bins (theta modulation index SD=.42), the lack of statistical significance is not surprising. During the first two time bins, infants were still deeply engaged with the video and not ready to orient away from ongoing to incoming stimulation. Nonetheless, the directionality of the results supports the evidence reported in the manuscript.

*Association with visual sensory seeking at follow-up*

To further support results of the associations with the ITSP visual sensory seeking scores at 10 months, an additional set of analyses was run with data from the follow-up ITSP that parents completed online six months after their infant participated to the study. The follow-up ITSP was returned for 39 of the 43 infants who contributed to the EEG analyses at 10 months (18 females, mean age = 16 months and 18 days, SD=37 days). Replication at a later time point would increase confidence in our results, given that parents’ ability to report on their infant’s sensory behaviour is dependent on the child’s developmental stage (Stone & Hogan, 1993; Baranek, 1999). Similarly, some of the psychometric properties of the ITSP improve with the infant’s developmental stage (Eeles et al., 2013).

Following the same analytical pipeline reported in the manuscript, we first replicated the significant association between the P1 modulation index and the visual sensory seeking scores at 16 months, (*r_s_* (22) = -.415, *p* = .022). Secondly, we replicated the significant association between the residuals of a regression with the theta modulation index as predictor and the P1 modulation index as outcome and the visual sensory seeking scores at 16 months, (*r_s_* (22) = -.388, *p* = .031) (see Figure 7). Similar to the 10-month results, also for the follow-up analyses, the item most strongly correlating with the EEG measures was item 20, which asks if the child prefers fast-paced, brightly coloured TV shows (see Table 2).

We also investigated this association in those 15 infants whose parents did not report TV exposure at 10 months. In this sub-group, significant associations emerged between ITSP visual sensory seeking at 16 months and P1 modulation index, *r_s_* (9) = -.553, *p* = .039; and regression residuals, *r_s_* (9) = -.659, *p* = .014. In both cases, the item most strongly correlating with these measures was item 20: for P1 modulation index, *r_s_* (9) = -.465, *p* = .075; for regression residuals, *r_s_* (9) = -.600, *p* = .025. This analysis rules out the possibility of reverse causation for the present dataset, i.e. that TV exposure at 10 months may drive information processing biases and reinforces our hypothesis that it is infants’ information processing bias that explains concurrent and later individual differences in visual sensory seeking.

Overall, results from this additional set of analyses confirm the evidence reported in the manuscript and support the idea that individual differences in information prioritization can account for alternative parent-reported visual seeking profiles both concurrently and longitudinally.

----Insert Figure 7 about here----

----Insert Table 2 about here----

**Figure list**

**Regression residuals**

**ITSP Visual Seeking score (16m)**

**Low seeking**

**High seeking**

***Rho* = -.388**

**Figure 7. Scatterplot illustrating the association between the ITSP visual seeking scores at 16 months and the residuals of a regression with the theta modulation index as predictor and the P1 modulation index as outcome. The statistical significance between the variables measured at 10 months was replicated at a later time point, i.e. 16 months.**

**Table list**

**Table 2. Cross-correlation table between the ITSP items contributing to the visual sensory seeking score at 10 and 16 months and the EEG measures (Spearman rho, **p* < .05).**

| ITSP 10m | Item 14  (N=29) | Item 15  (N=29) | Item 19  (N=29) | | Item 20  (N=14) |
| --- | --- | --- | --- | --- | --- |
| P1 modulation index | .118 | .085 | -.074 | -.191 | |
| Regression residuals | .045 | -.032 | -.054 | -.267 | |

| ITSP 16m | Item 14  (N=24) | Item 15  (N=24) | Item 19  (N=24) | Item 20  (N=24) |
| --- | --- | --- | --- | --- |
| P1 modulation index | -.211 | -.211 | -.133 | -.369* |
| Regression residuals | -.119 | -.204 | -.117 | -.381* |
